# Supplementary material for: Vms1p is a release factor for the ribosome-associated quality control complex
Source: Nat Commun. 2018 Jun 6;9:2197. doi: 10.1038/s41467-018-04564-3 (PMC5989216; doi:10.1038/s41467-018-04564-3)
Supplement: Supplementary file 1 — Supplementary Information [file 41467_2018_4564_MOESM1_ESM.pdf]

Supplementary Information

Vms1p is a release factor for the Ribosome-associated Quality control Complex

Zurita-Rendon et al.

Supplementary Figure 1

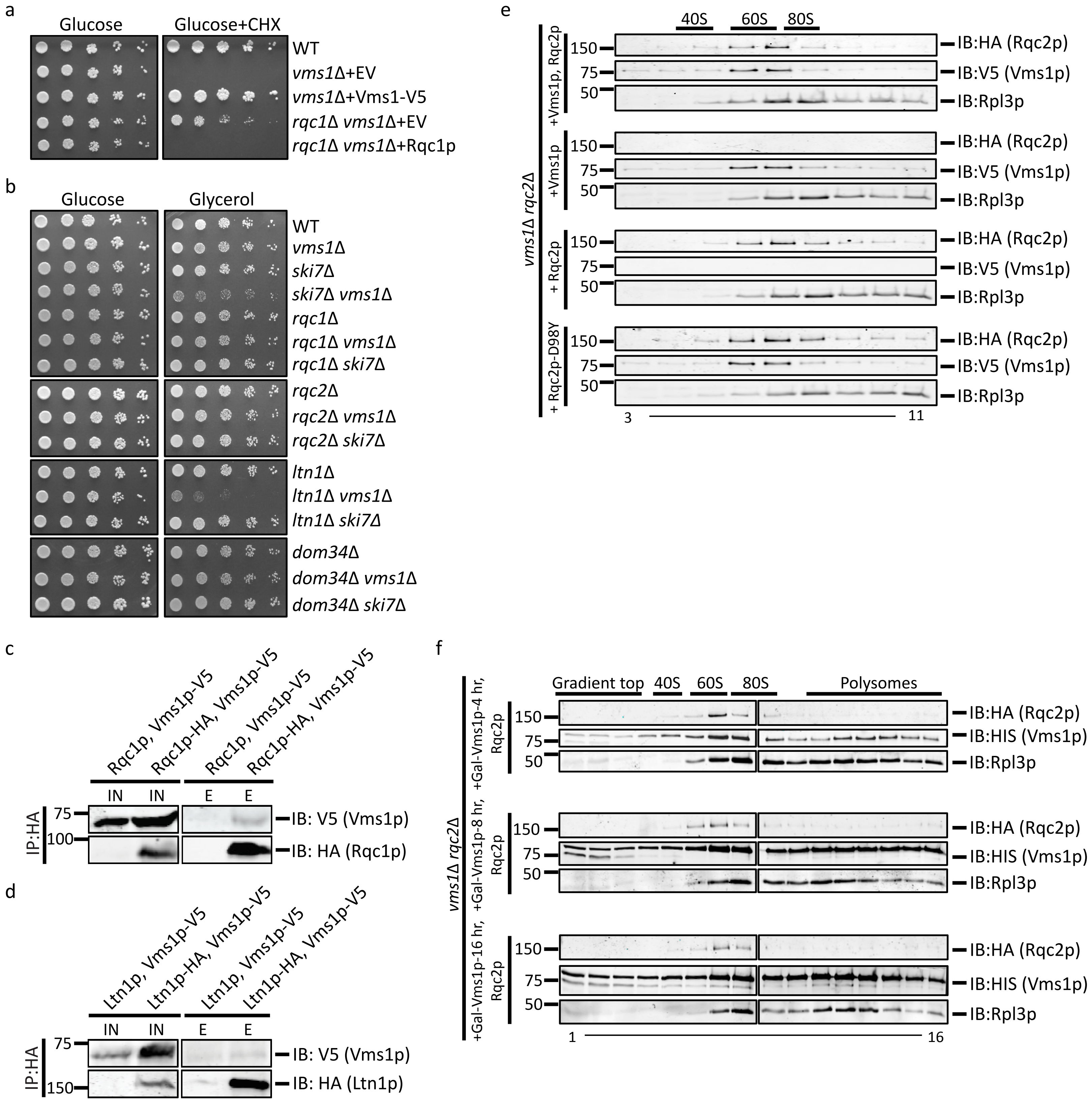

Supplementary Figure 1.

(a) Serial dilutions of indicated strains were spotted on media containing glucose or glucose supplemented with cycloheximide (CHX) and grown for 2 or 3 days, respectively.

(b) Serial dilutions of the indicated strains were spotted on medium containing glucose or glycerol and grown for 2 or 3 days, respectively.

(c) Immunoprecipitations using anti-HA antibody in the *rqc1*Δ *vms1*Δ strain expressing Rqc1p and Vms1p-V5 (control) or Rqc1p-HA and Vms1p-V5. Immunoblotting of HA and V5 were used to identify Rqc1p and Vms1p, respectively.

(d) Immunoprecipitations using anti-HA antibody in the *ltn1*Δ *vms1*Δ strain expressing Ltn1p and Vms1p-V5 (control) or Ltn1p-HA and Vms1p-V5. Immunoblotting of HA and V5 were used to identify Ltn1p and Vms1p, respectively.

(e) Polysome profiles of whole cell extracts from the *vms1*Δ *rqc2*Δ strain expressing Rqc2p-HA and Vms1p-V5, Vms1p-V5, Rqc2p-HA or the Rqc2p CAT-tailing-defective mutant Rqc2p-D98Y from top to bottom, respectively. Strains were treated with CHX prior to fractionation by sucrose density centrifugation. Chromatographic analysis (A_{254}) was used to determine the distribution of the 40S, 60S, 80S and polysome content of the 16 collected fractions. Immunoblot analysis was performed only on fractions 3-11. The distribution of the 60S subunit was confirmed by immunoblotting of the ribosomal subunit, Rpl3p. Immunoblotting of HA and V5 was used to detect Rqc2p and Vms1p, respectively.

(f) Polysome profiles of whole cell extracts from the *vms1*Δ *rqc2*Δ strain expressing Rqc2p-HA and Vms1p-V5 under the *GAL*-inducible promoter after galactose induction for 4, 8 and 16 hr from top to bottom, respectively. Chromatographic analysis (A_{254}) was used to determine the distribution of the 40S, 60S, 80S and polysome content of the 16 collected fractions. The distribution of the 60S subunit was confirmed by immunoblotting of the ribosomal subunit, Rpl3p. Immunoblotting of HA and V5 was used to detect Rqc2p and Vms1p, respectively.

Supplementary Figure 2

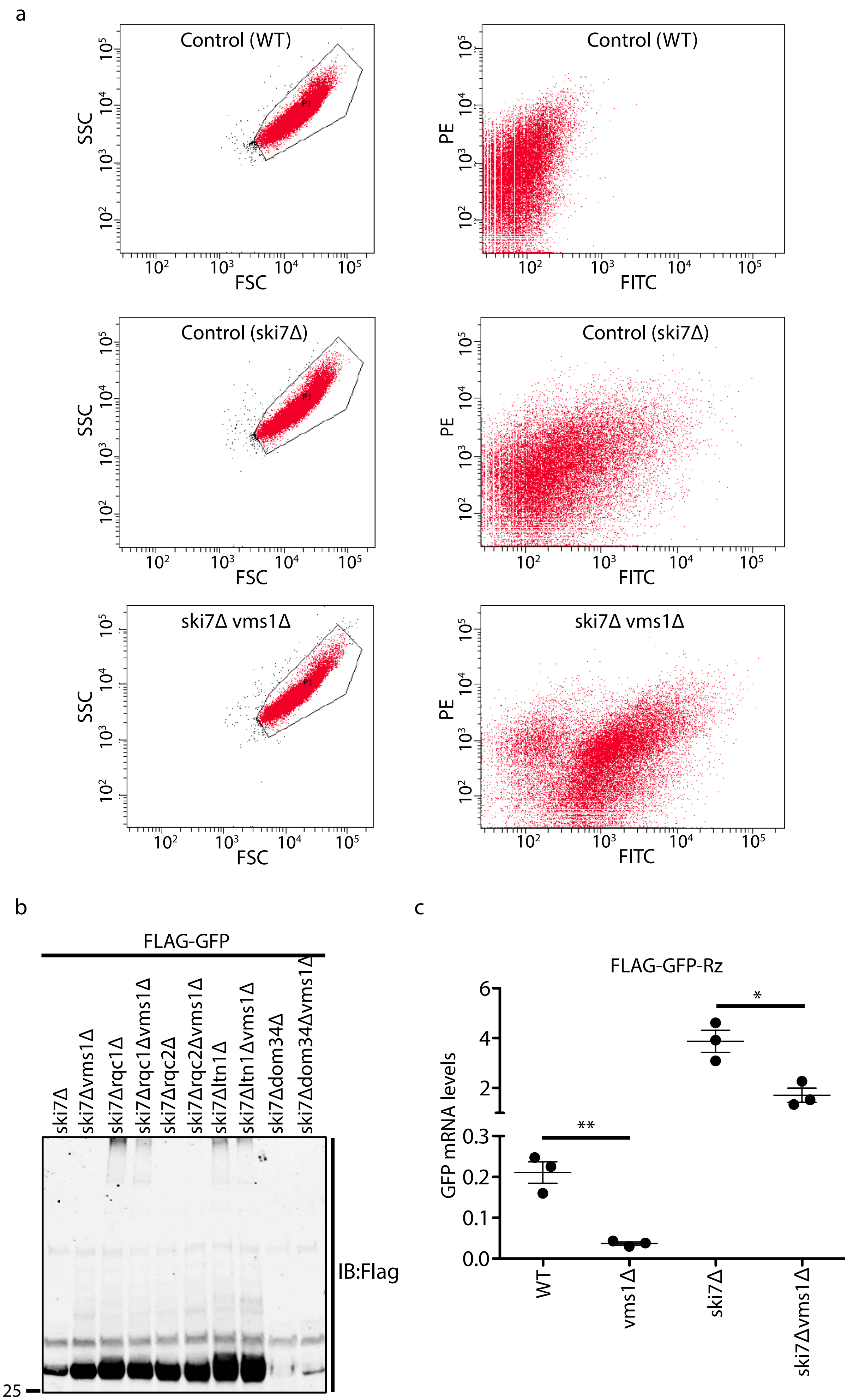

Supplementary Figure 2.

(a) Gating strategy for analyzing FLAG-GFP positive cells. Panel shows gating parameters for collection of total GFP intensity, excluding cellular debris in WT, *ski7*Δ and *ski7*Δ*vms1*Δ. SSC, Side Scatter light; FSC, Forward Scatter light; PE, phycoerythrin; FITC, Fluorescein isothiocyanate. PE was plotted but not analyzed in this study.

(b) Immunoblot analysis of whole cell extracts from the indicated strains expressing the FLAG-GFP^{Rz} construct (same as in Fig. 2). Immunoblotting of Flag (overexposed) was used to detect the accumulation of aggregates in the stacking portion of the gel.

(c) qRT-PCR analysis of the indicated strains expressing the FLAG-GFP^{Rz} construct (n=3, data are mean ± s.e.m. $^{**}P < 0.002$ and $^{*}P < 0.01$, P-value was calculated using unpaired Student's t-test).

Supplementary Figure 3

a

Description	Chain	Z score	RMSD	lali	%ID
Elongation factor I-alpha	3vmf-B	6.9	3.4	111	6
Eukaryotic peptide chain release factor 1	3e1y -C	6.8	4.1	109	4
Eukaryotic peptide chain release factor	1dt9-A	6.6	3.1	105	4
Peptide chain release factor 1	4af1 -A	6.5	4.0	116	8
Dom34	2vgn -A	6.5	5.0	115	7
Pelota	3obw-A	6.4	3.9	103	13
Dom34	3izq -0	6.0	4.0	122	6
Elongation factor 1-alpha	3agj -B	6.0	4.1	121	7
Pelota	3oby -A	5.9	4.3	120	4
Ribonuclease E	2c0b -L	5.9	3.1	92	11

b

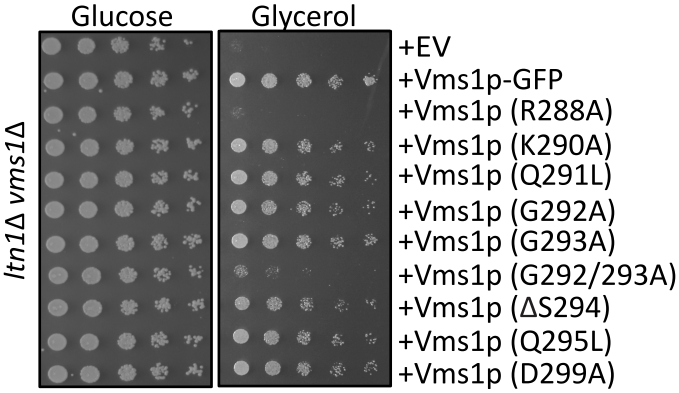

c

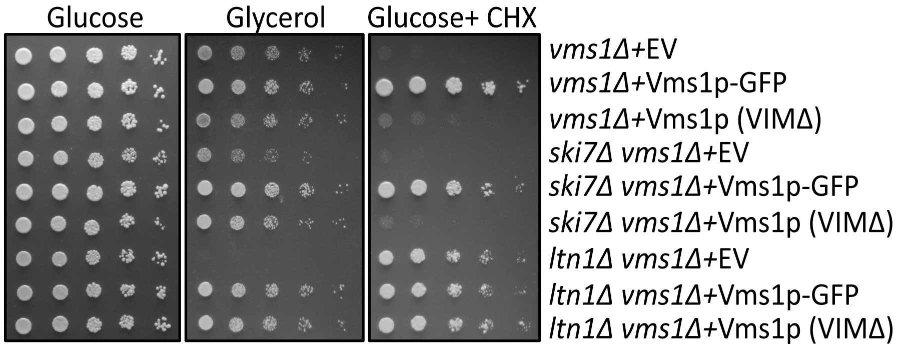

d

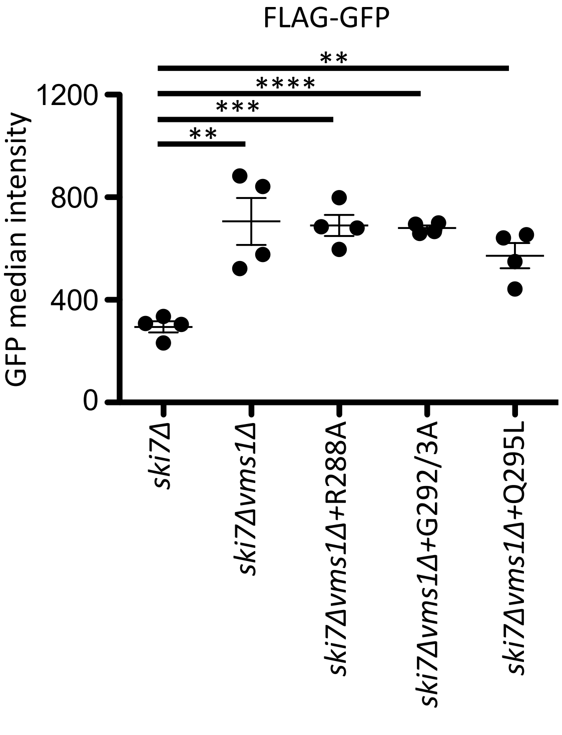

e

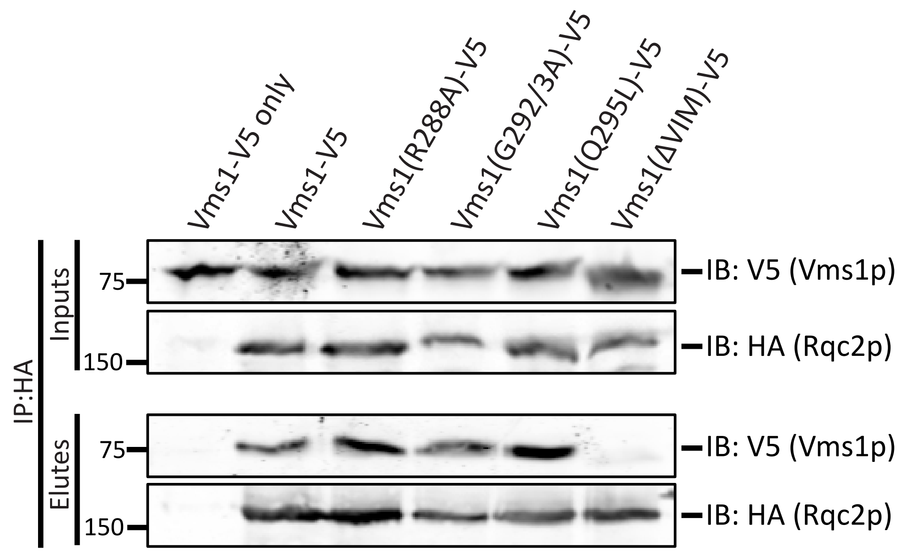

Supplementary Figure 3.

(a) Similar structures to the Vms1p MTD/eRFL returned from the Dali server[1]. Z-score indicates degree of structural similarity, with above 2 being a similar fold. lali, number of aligned residues; %ID, percent identical residues.

(b) Serial dilutions of *ltn1*Δ *vms1*Δ cells with the indicated plasmids were spotted on synthetic media supplemented with glucose or glycerol.

(c) Serial dilutions of indicated strains were spotted on glucose, glycerol and glucose supplemented with cycloheximide (CHX) and grown for 2 or 3 days, respectively.

(d) Flow cytometry quantifications of FLAG-GFP accumulation in the indicated strains. Median GFP intensity values (n=4, data are mean ± s.e.m. $^{**}P < 0.004$, $^{***}P < 0.0002$, $^{****}P < 0.0001$, P-value was calculated using unpaired Student's t-test).

(e) Immunoprecipitation using the anti-HA antibody in the *rqc2*Δ *vms1*Δ strain expressing Rqc2p and Vms1p-V5 (control); Rqc2p-HA and Vms1p-V5; or Rqc2p-HA and Vms1p-V5 mutants. Immunoblotting of HA and V5 was used to identify Rqc2p and Vms1p, respectively.

Supplementary Figure 4

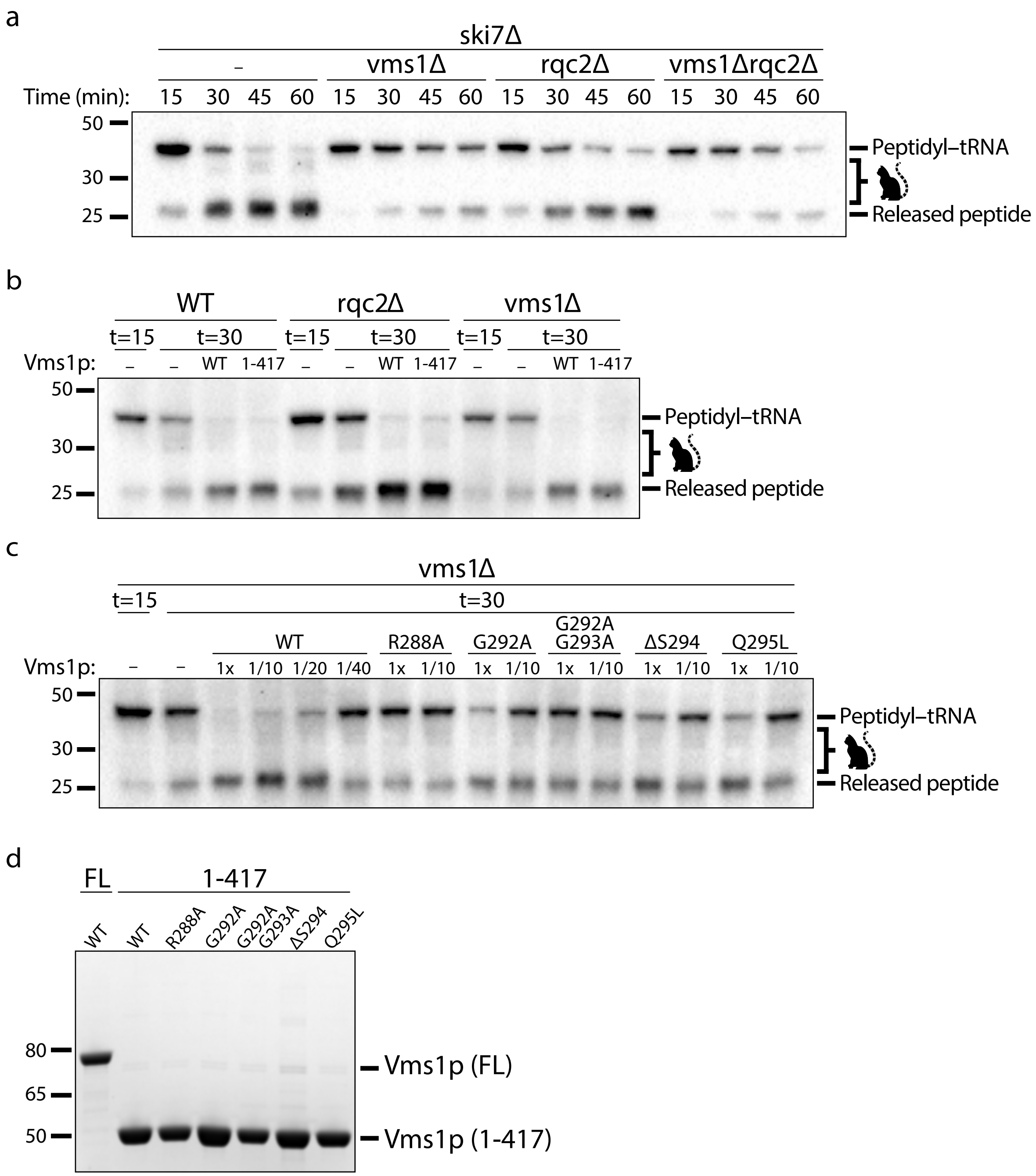

Supplementary Figure 4.

(a) Time courses of *S. cerevisiae* in vitro translation (ScIVT) reactions prepared with a truncated mRNA (lacking a stop codon). Extract genotypes are indicated above. Peptides that have been CAT-tailed and released are denoted by a cat icon.

(b) ScIVT reactions prepared as in (a) with WT, *rqc2Δ*, or *vms1Δ* extracts. At *t*=15, buffer (-) or pure protein (4.2 μM final) was added. FL = Full Length Vms1; 1-417 = N-terminus through eRF1-like domain.

(c) ScIVT reactions prepared as in (a) with a *vms1Δ* extract. At *t*=15, buffer, WT(1-417), or mutant(1-417) protein was added (see Methods).

(d) Coomassie staining of purified Vms1 proteins used in ScIVT rescue experiments. FL = Full Length; 1-417 = N-terminus through eRF1-like domain.

Supplementary Figure 5

a

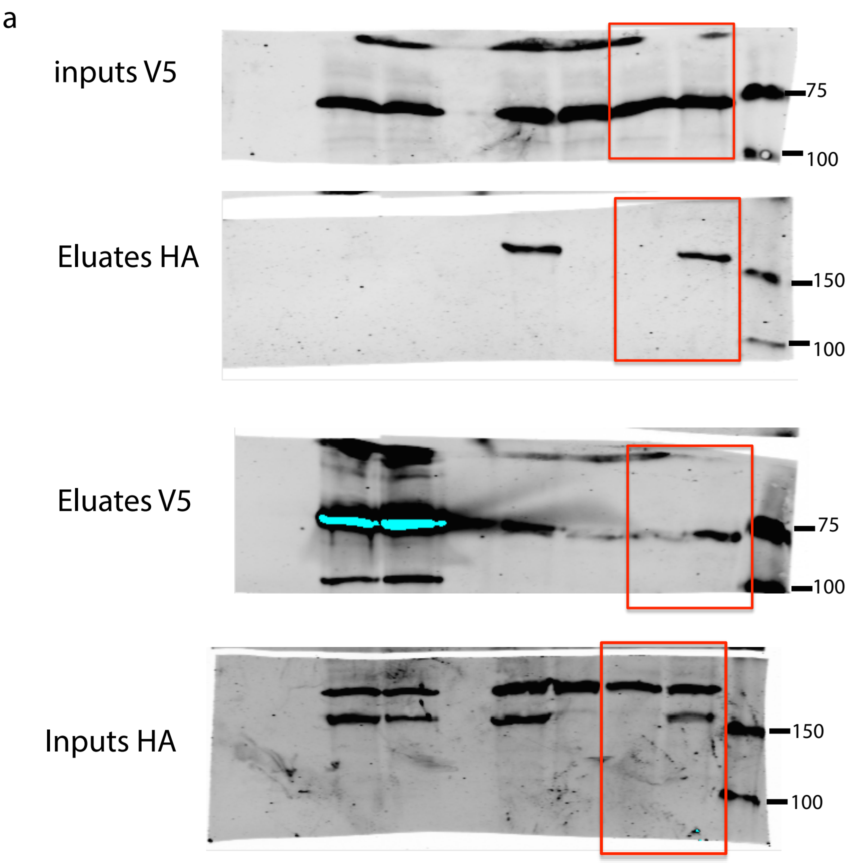

b

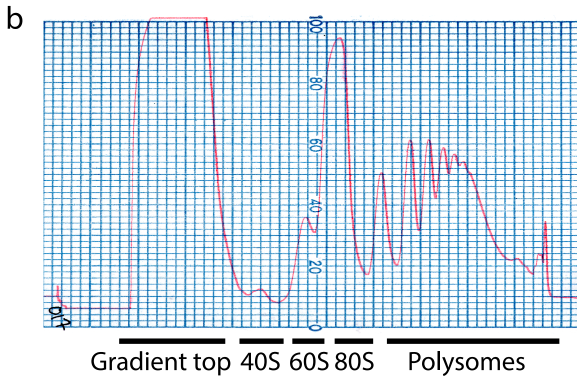

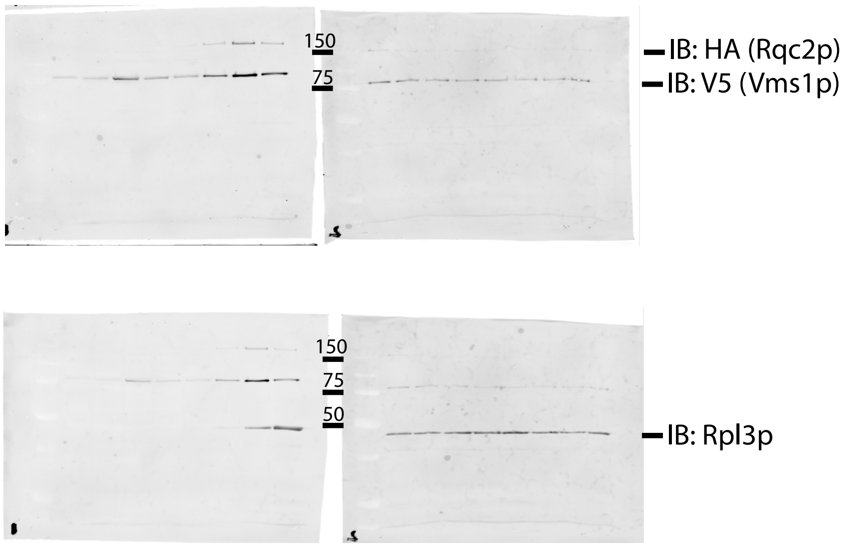

Supplementary Figure 5.

(a) Uncropped blots from figure 1c. Red boxes indicate the lanes used in the figure.

(b) Raw polysome trace and uncropped blots from figure 1d.

Supplementary Figure 6

a

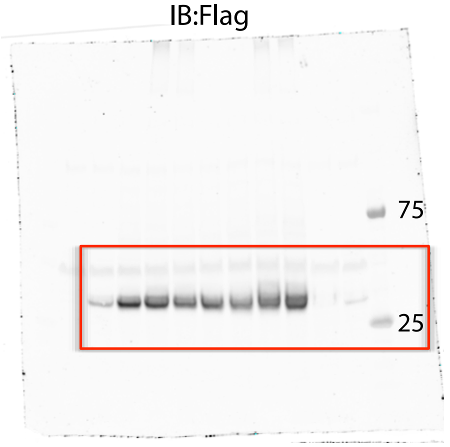

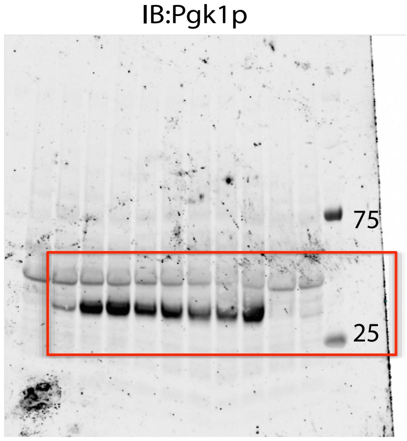

Supplementary Figure 6.

(a) Uncropped blots from figure 2c.

Supplementary Figure 7

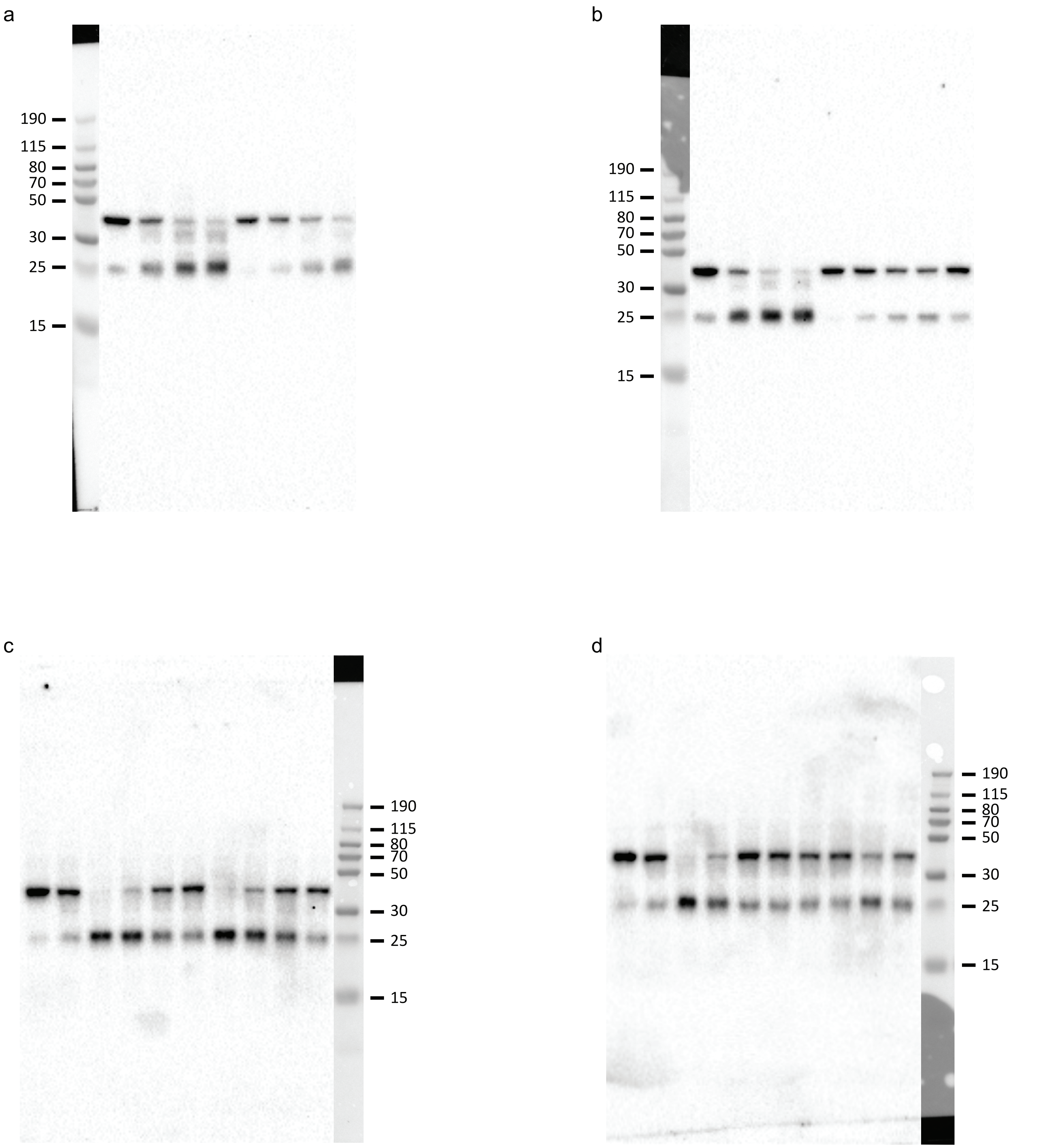

Supplementary Figure 7.

(a) Uncropped blot from figure 4a.

(b) Uncropped blot from figure 4b.

(c) Uncropped blot from figure 4c.

(d) Uncropped blot from figure 4d.

Supplementary References

1. Holm L, Rosenstrom P. Dali server: conservation mapping in 3D. *Nucleic Acids Res* **38**, W545-549 (2010).

Supplementary Table 1

**JR Library Name**	**Genotype (BY4741, a haploid strains)**
JRY2884	WT
JRY3377	*vms1::NatMx*
JRY3491	*ski7::HygroMx*
JRY3378	*ski7::HygroMx vms1::NatMx*
JRY3380	*rqc1::KanMx*
JRY3383	*rqc1::KanMx vms1::NatMx*
JRY3382	*rqc1::KanMx ski7::HygroMx*
JRY3386	*rqc1::KanMx vms1::NatMx ski7::HygroMx*
JRY3388	*rqc2::KanMx*
JRY3392	*rqc2::KanMx vms1::NatMx*
JRY3390	*rqc2::KanMx ski7::HygroMx*
JRY3394	*rqc2::KanMx vms1::NatMx ski7::HygroMx*
JRY3401	*ltn1::KanMx*
JRY3405	*ltn1::KanMx vms1::NatMx*
JRY3403	*ltn1::KanMx ski7::HygroMx*
JRY3503	*ltn1::KanMx vms1::NatMx ski7::HygroMx*
JRY3410	*dom34::KanMx*
JRY3415	*dom34::KanMx vms1::NatMx*
JRY3412	*dom34::KanMx ski7::HygroMx*
JRY3416	*dom34::KanMx vms1::NatMx ski7::HygroMx*
JRY1734	*pep4Δ::HIS3 prb1Δ::LEU2 bar1Δ::HISG lys2::GAL1/10-GAL4*

Supplementary Table 2

Vector backbone	Construct	JR library name
pRS413	EV-HIS3	189
pRS415	EV-LEU2	191
pRS416	EV-URA3	192
pRS416	GFP	1415a
pRS415, 416	pVMS1-VMS1-V5	13862, 13863
pRS415, 416	pVMS1-VMS1-GFP	3462D, 10703B
pRS416	pVMS1-VMS1-VIMΔ-GFP	10720B
pRS416	pVMS1-VMS1-VIMΔ-V5	13864
pRS416	pVMS1-VMS1-H279A-V5	13868
pRS416	pVMS1-VMS1-H283A-V5	13869
pRS416	pVMS1-VMS1-R284A-V5	13870
pRS416	pVMS1-VMS1-Y285A-V5	13871
pRS416	pVMS1-VMS1-T286A-V5	13872
pRS416	pVMS1-VMS1-R288A-GFP	13873
pRS416	pVMS1-VMS1-K290A-GFP	13874
pRS416	pVMS1-VMS1-Q291L-GFP	13875
pRS416	pVMS1-VMS1-G292A-GFP	13876
pRS416	pVMS1-VMS1-G293A-GFP	13877
pRS416	pVMS1-VMS1-G292/93A-GFP	13878
pRS416	pVMS1-VMS1-ΔS294-GFP	13879
pRS416	pVMS1-VMS1-Q295L-GFP	13880
pRS416	pVMS1-VMS1-D299A-GFP	13881
pRS416	pVMS1-VMS1-R288A-V5	13865
pRS416	pVMS1-VMS1-G292/293A-V5	13866
pRS416	pVMS1-VMS1-Q295L-V5	13867
pRS415	pTP1-mtRFP	3862A
pRS416	pRQC1-RQC1	13852
pRS416	pRQC1-RQC1-2HA	13853
pRS415	pRQC2-RQC2	13854
pRS415	pRQC2-RQC2-2HA	13855
pRS415	pRQC2-RQC2-D98Y	13856
pRS416	pLTN1-LTN1	13857
pRS416	pLTN1-LTN1-2HA	13858
pRS416	pLTN1-LTN1-W1542E	13859
pRS416	pGPD-3XFLAG-6XHIS-GFP-RZ	13860
pRS413	pFUM1-FUM1-3XFLAG-6XHIS-GFP-RZ	13861
pRS416	PGAL1-12HIS-VMS1	10755F
pRS416	PGAL1-12HIS-VMS1-R288A	13882

Supplementary Table 2

pRS416	PGAL1-12HIS-VMS1-G292A	13883
pRS416	PGAL1-12HIS-VMS1-G292/293A	13884
pRS416	PGAL1-12HIS-VMS1-ΔS294	13885
pRS416	PGAL1-12HIS-VMS1-Q295L	13886
pRS416	PGAL1-12HIS-VMS1(1-417)	10755B
pRS416	PGAL1-12HIS-VMS1(1-417, R288A)	13887
pRS416	PGAL1-12HIS-VMS1-(1-417, G292A)	13888
pRS416	PGAL1-12HIS-VMS1-(1-417, G292/293A)	13889
pRS416	PGAL1-12HIS-VMS1-(1-417, ΔS294)	13890
pRS416	PGAL1-12HIS-VMS1-(1-417, Q295L)	13891
